# Electrophysiological Evidence Reveals Differences between the Recognition of Microexpressions and Macroexpressions

**DOI:** 10.3389/fpsyg.2016.01346

**Published:** 2016-08-31

**Authors:** Xunbing Shen, Qi Wu, Ke Zhao, Xiaolan Fu

**Affiliations:** ^1^Department of Psychology, Jiangxi University of Traditional Chinese MedicineNanchang, China; ^2^State Key Laboratory of Brain and Cognitive Science, Institute of Psychology, Chinese Academy of SciencesBeijing, China; ^3^Department of Psychology, Hunan Normal UniversityChangsha, China

**Keywords:** microexpression, macroexpression, recognition, EEG/ERPs, ERSP, sLORETA

## Abstract

Microexpressions are fleeting facial expressions that are important for judging people’s true emotions. Little is known about the neural mechanisms underlying the recognition of microexpressions (with duration of less than 200 ms) and macroexpressions (with duration of greater than 200 ms). We used an affective priming paradigm in which a picture of a facial expression is the prime and an emotional word is the target, and electroencephalogram (EEG) and event-related potentials (ERPs) to examine neural activities associated with recognizing microexpressions and macroexpressions. The results showed that there were significant main effects of duration and valence for N170/vertex positive potential. The main effect of congruence for N400 is also significant. Further, sLORETA showed that the brain regions responsible for these significant differences included the inferior temporal gyrus and widespread regions of the frontal lobe. Furthermore, the results suggested that the left hemisphere was more involved than the right hemisphere in processing a microexpression. The main effect of duration for the event-related spectral perturbation (ERSP) was significant, and the theta oscillations (4 to 8 Hz) increased in recognizing expressions with a duration of 40 ms compared with 300 ms. Thus, there are different EEG/ERPs neural mechanisms for recognizing microexpressions compared to recognizing macroexpressions.

## Introduction

Facial expressions serve important social functions, and the recognition of emotional facial expressions is vital for everyday life (Niedenthal and Brauer, 2012). However, emotion is not necessarily displayed on the face at all times. In a number of interpersonal situations, people hide, disguise, or inhibit their true feelings (Ekman, 1971), leading to partial or very rapid production of expressions of emotion, which are called microexpressions (Ekman and Friesen, 1969; Bhushan, 2015).

A microexpression is a facial expression that lasts between 1/25 and 1/5 of a second, revealing an emotion that a person is trying to conceal (Ekman and Friesen, 1969; Ekman, 1992, 2003; Porter and ten Brinke, 2008). A microexpression resembles one of the universal emotions: disgust, anger, fear, sadness, happiness, or surprise. Microexpressions usually occur in high-stakes situations in which people have something valuable to gain or lose (Ekman et al., 1992). According to Ekman (2009), microexpressions are believed to reflect a person’s true intent, especially one of a hostile nature. Therefore, microexpressions can provide an essential behavioral clue for lie detection and can be employed to detect a dangerous demeanor (Metzinger, 2006; Schubert, 2006; Weinberger, 2010).

Little is known regarding the characteristics that differentiate microexpressions and macroexpressions. The most important difference between microexpressions and macroexpressions is their duration (Svetieva, 2014). However, there are different estimates of the duration of microexpressions (Shen et al., 2012). According to Shen et al. (2012), there are at least six estimates of the duration of microexpressions, and 200 ms duration can be used as a boundary for differentiating microexpressions and macroexpressions. However, it is unclear whether there are different neural indicators for recognizing expressions with durations of less than 200 ms and those with durations of longer than 200 ms. If microexpressions and macroexpressions are qualitatively different (from the viewpoint of the perceiver, they should be recognized as different objects), then it can be expected that there are different brain mechanisms for processing facial expressions with a duration shorter than or longer than the duration boundary (200 ms).

Thus, we aimed to investigate the neural mechanisms for recognizing expressions with different durations, which can aid in the evidence-based separation of microexpressions from macroexpressions (i.e., to determine the boundary between microexpressions and macroexpressions). In other words, if differences in the neural characteristics that recognize one group of expressions with one kinds of duration and another group of expressions with other kinds of duration exist, we can say that the two groups of expressions are different. If we can find the discrepancy in the electroencephalogram (EEG)/event-related potentials (ERPs) between expressions with different durations, we can divide expressions with different EEG/ERPs characteristics into two groups. One group can be called microexpressions (with a short duration), and the other can be called macroexpressions (with a longer duration). Given the behavioral difference in recognition of microexpressions and macroexpressions and the disagreement regarding the conceptual definition for the duration of a microexpression, we seek to find electrophysiological evidence of the boundary (200 ms) that separates microexpressions from macroexpressions.

The EEG can indicate the characteristic temporal, spatial, and spectral signatures of specific cognitive processes. We explored the EEG activities during recognizing expressions with different duration (40, 120, 200, and 300 ms) to examine whether there is a turning point near 200 ms as indicated by the EEG measurements. Two different objects or ideas should only be thought of as separate entities when they have a number of differing characteristics. If neural differences are present before and after the turning point (e.g., 200 ms), then we can safely say that the duration of the conceptual definition of a microexpression is less than the turning point (the upper limit of microexpression duration). As there are different behavioral characteristics in the recognition of expressions with a duration of less than 200 ms and expressions with a duration longer than 200 ms (Shen et al., 2012), we hypothesized that recognizing expressions with a duration of less than 200 ms and expressions with a duration of longer than 200 ms will show different EEG characteristics (i.e., amplitude, oscillatory dynamics, and source location). Consequently, there should be different brain mechanisms for recognizing microexpressions and macroexpressions. Hence, the present study aimed to provide evidence for separating microexpressions and macroexpressions by investigating EEG/ERPs and synchronized oscillatory activity.

We used an affective priming paradigm, in which a picture of a facial expression is the prime and an emotional word is the target. Meanwhile, we mainly focused on the ERPs components of N400 and N170. The N400 can be produced not only in instances of semantic mismatch but also in other incongruous meaningful stimuli, such as words and faces. The effects of the N400 can also be observed in response to line drawings, pictures, and faces when primed by single items or sentence contexts, but not in the absence of priming (Kutas and Federmeier, 2011). In a pilot experiment, we found that the N400 could be elicited in the expression – emotional word priming paradigm. This N400 amplitude is more negative for incongruent than for congruent emotional content of face-word pairs. To produce a greater N400 effect, an incongruent condition in the experiment that elicits a greater negative-going wave than does the congruent condition should also be present. Therefore, we employed pictures of facial expressions (happy, fearful, and neutral) as priming stimuli and emotional words (positive and negative) as targets. Consequently, there were three conditions (congruent, incongruent, and control) with respect to the congruence of emotional valence.

Expressions can have different durations. There are expressions with short duration (e.g., less than 200 ms). If we recognize them as the same because of the limited time to process them, then during conditions of short duration for recognizing expressions, there is no congruence or incongruence due to expressions with different short durations being observed as the same by the participants. On the contrary, expressions with different long durations (e.g., greater than 200 ms) will appear to be different to the participants due to the extensive time for processing, which can result in congruence and incongruence. Thus, there will be no effect of congruence at the short duration; however, the effect of congruence at the long duration will be significant. To put it another way, when the presentation of an expression is transient, there is no top-down influences on the recognition of the expression. Consequently, there should be no difference between the congruent and incongruent conditions. Only when the duration of the expression is sufficiently long do the participants engage in top-down processing and recognize the expressions differently, which results in the congruent and incongruent conditions. Therefore, we could expect that there will be a significant relationship between duration and congruence while measuring N400 effects which reflect the top-down influences (Newman and Connolly, 2009). If we could extensively process expressions with short duration (less than 200 ms), meaning there was no difference between expressions with short and long duration, then there would be no relationship between duration and congruence.

For facial processing, one of the most prominent components in the ERPs is the N170 (Rossion and Jacques, 2011), and the face-sensitive N170 is modified by facial expressions of emotion (Batty and Taylor, 2003; Righart and De Gelder, 2008). As noted by Joyce and Rossion (2005), Eimer (2011), the N170 may be a vertex positive potential (VPP), resulting from changing the reference electrodes from the mastoid to the common average reference. The N170/VPP may be a valuable tool for studying the cognitive and neurobiological mechanisms underlying expression recognition. If there is a turning point in accuracy for recognizing expressions with different durations (i.e., there is a duration boundary for microexpressions and macroexpressions; see Shen et al., 2012), then we can expect that the main effect of duration will be significant. Specifically, there should be a significant difference between an expression with duration of less than 200 ms (microexpression) and an expression with duration of longer than 200 ms (macroexpression) while measuring N170/VPP. That is, there should be two groups of N170/VPP, one for microexpressions and one for macroexpressions (which can lead to a conclusion that expressions with short and long durations fall into two different categories).

It is worth noting that the priming paradigm provides an avenue for studying expression perception and recognition, which is appropriate for our aims. First, we wanted to investigate the effect of duration on the ERPs of expression recognition; when the duration of the expression is longer (macroexpression), the valence of the expression will be processed and the later processing of the emotional word will be facilitated or inhibited. Consequently, the N400 will reflect the facilitated or inhibited effect, i.e., there should be a smaller N400 when the valence of the expression and the emotional word are congruent. However, when the duration of expression is shorter (microexpression) the valence of the expression may not be fully processed, and there may be no facilitated or inhibited effect. Therefore, the N400 for the processing of microexpressions should not be affected, regardless of the congruence of the emotional valence. Second, this paradigm offers insights into the time course of the perception and the recognition of microexpressions and macroexpressions while measuring N170/ VPP.

The information regarding oscillatory dynamics from the EEG signal is largely lost by the time-locked averaging of single trials in the traditional ERPs approach. Researching functional correlates of brain oscillations is an important current trend in neuroscience. The traditional spectral analysis cannot fully address the issue of rapidly changing neural oscillations. Time–frequency analysis of an EEG allows researchers to study the changes of the signal spectrum over time, taking into account the power (or amplitude) of the EEG signal at a given frequency as well as changes in the phase or latency (Buzsáki, 2006; Roach and Mathalon, 2008; Güntekin and Baȿsar, 2014). Some recent studies investigated the mechanisms of perception and categorization of emotional stimuli through brain oscillatory activity extracted from EEG signals (Keil, 2013). Oscillatory dynamics of theta, alpha, beta, and gamma bands, and the interplay of these frequencies, relates to the processing of emotional stimuli (Güntekin and Baȿsar, 2014). Furthermore, some EEG studies show that the theta band activities, which are associated with subcortical brain regions and are considered to be the fingerprint of all limbic structures, are involved in affective processes (Knyazev and Slobodskaya, 2003). Meanwhile, theta band activity was observed during emotional stimulus presentation and it was associated with emotion comprehension (Balconi and Pozzoli, 2007). Therefore, this study mainly explores the dynamic oscillatory patterns of theta bands activities in the EEG signal while recognizing microexpressions and macroexpressions.

Previous studies (Esslen et al., 2004; Costa et al., 2014) had found that different emotional conditions had different activation patterns in different brain regions by using the low resolution brain electromagnetic tomography. In the current study, we also employed Standardized Low Resolution Brain Electromagnetic Tomography (sLORETA; Fuchs et al., 2002; Pascual-Marqui, 2002; Jurcak et al., 2007) to identify brain regions involved in recognizing expressions with long and short durations.

## Materials and Methods

All experimental protocols were approved by the Institutional Review Board of the Institute of Psychology, Chinese Academy of Sciences. The methods were carried out in accordance with the approved guidelines.

### Participants

Sixteen paid volunteers (8 female, ages 20 to 25 years, mean age = 22.3; 8 male, ages 22 to 24, mean age = 22.6) with no history of neurological injury or disorder were recruited from local college campuses. They gave written informed consent before participating. All participants had normal or corrected-to-normal vision and were predominantly right-handed (self-reported). Data from four participants containing too many artifacts were excluded from the analysis (including one participant with higher score of SDS, see the Results section), and the final analyses were conducted on twelve participants (7 female, mean age ± SD: 22.4 ± 1.4 years).

### Stimuli and Experimental Design

The pictures of faces consisted of 10 different individuals displaying fear (negative), happiness (positive) or a neutral expression; a total of 30 pictures of facial expressions were selected from 10 models taken from the Pictures of Facial Affect (POFA^1^). The emotional words consisted of 50 positive and 50 negative Chinese words selected from Wang and Fu (2011). The picture stimuli were 200 pixels × 300 pixels, and the word stimuli were 100 pixels × 150 pixels.

The stimuli were presented at a viewing distance of approximately 80 cm and displayed at a moderate contrast (black letters on a silver-gray background) in the center of a 17-inch computer screen with a refresh rate of 60 Hz. The experimental design was as follows: 4 durations (40, 120, 200, and 300 ms) × 3 congruencies (Congruence, Incongruence, and Control).

### Procedure

The participants were seated in a comfortable armchair in a dimly lit, sound damped booth. Emotional faces and words were presented using a priming paradigm. Subjects were asked to remember all of the content displayed on the screen to focus their attention on the task and to ensure the depth of processing of the words and pictures. No other tasks were imposed on the subjects during the ERPs recordings to avoid confounding the EEG for emotion processing with electrophysiological activity associated with motion for response selection and response execution. The experiment was divided into four blocks according to duration, with each lasting approximately 15 min. At the end of each block, the participants were given a test of recognition. After each block, the subjects were allowed to rest for 2 min. After the EEG recordings, each subject was asked to rate their mood using the Chinese version of the Zung Self Rating Anxiety and Depression Scales (SAS), SDS, selected from Wang et al. (1993).

Stimuli appeared one at a time in trials consisting of pictures of faces and emotional words. Four blocks were divided by the duration of exposure to the pictures of faces, which were 40, 120, 200, and 300 ms. Each trial consisted of a succession of stimuli: a fixation (with a duation randomly selected from 300 to 500 ms), a facial expression picture expressing one of the three emotions (with duration of 40, 120, 200, or 300 ms), a blank screen (the range in duration from 100 to 400 ms), one of the positive or negative emotional words (with duration of 1000 ms), and an interval (the range in duration from 1200 to 1500 ms). There were 300 trials per block. The order of presentation of the four blocks was randomized between subjects. The trial order within each block was randomized. At the end of each block, there was a recognition task (the participants had to judge whether some items including pictures and words were presented before), and the accuracy was measured to monitor the degree of cooperation of the participants. A break of approximately 2 min controlled by the participants separated each successive block.

### Electrophysiological Recording and Analyses

#### EEG/ERPs Acquisition and Analyses

Data were acquired from a 32-channel NuAmps Quickcap, 40-channel NuAmps DC amplifier and Scan 4.5 Acquisition Software (Compumedics Neuroscan, Inc., Charlotte, NC, USA). The EEG data were recorded from 30 scalp sites (Fp1, Fp2, F7, F8, F3, F4, FT7, FT8, T3, T4, FC3, FC4, C3, C4, CP3, CP4, TP7, TP8, T5, T6, P3, P4, O1, O2, Fz, FCz, Cz, CPz, Pz, and Oz). The NuAmps (Model 7181) amplifier had a fixed range of ±130 mV sampled with a 22-bit A/D converter, where the least significant bit was 0.063 μV. The impedance of the recording electrodes was monitored for each subject prior to data collection, and the threshold was always kept below 5 KΩ. The amplifier was set at a gain of 19, with a sampling rate of 1000 Hz and with a signal band limited to 70 Hz. In addition, no notch filter was applied. The electro-oculograms (EOG) were measured to exclude them from the EEG recordings. Vertical EOG (VEOG) was recorded by electrodes 2 cm above and below the left eye and in line with the pupil. The horizontal EOG (HEOG) was recorded by electrodes placed 2 cm from the outer canthi of both eyes. The ground electrode was positioned 10 mm anterior to Fz. The right mastoid electrode (M2) was used as the reference for all recordings and all data were oﬄine re-referenced to a common average reference.

The EEG was later reconstructed into discrete, single-trial epochs. For analyzing the N170/VPP of facial expressions with different durations, an EEG epoch length of 400 ms was used, with a 100 ms pre-stimulus baseline and a 300 ms period, following the onset of the emotional faces. EEG epochs that exceeded ±100 μV were excluded, all trials were visually scanned for further artifacts generated by non-cerebral sources, and corrections were made for eye blinks. Participants had no fewer than 90 accepted epochs in any condition. The accepted epochs were recomputed to the average reference oﬄine and were baseline corrected. The ERPs were averaged separately for each experimental condition. For the averaged N170/VPP wave, a mean amplitude measure within a 140–200 ms time window from onset of the facial stimuli of each participant was provided. The mean amplitude of the N170/VPP then was analyzed by a repeated-measures analyses of variance (ANOVA), in which the factors Valence (positive, negative, and neutral) × Duration (40, 120, 200, and 300 ms) to the mean amplitude were compared.

Facial stimuli under the incongruent condition elicited greater centroparietal ERPs negativity than those under the congruent condition. We termed this negative-going waveform as N400. For this ERPs wave, the epoch length of 1000 ms was used, with a 200 ms pre-stimulus baseline and an 800 ms period, following the onset of the emotional words. A mean amplitude measure within a 350–500 ms time window from the emotional word stimulus onset was provided. The time window of the N400 was selected by visually inspecting, and it more closely resembled a conventional time window of N400. An ANOVA was performed on the N400 mean amplitude.

The N400 was typically maximal over the centro-parietal electrode sites. Therefore, electrodes Cz and CPz were selected for further N400 statistical analysis (one-way analysis of variance, ANOVA), which was carried out on the mean N400 amplitude measurements at the midline central (Cz) and parietal (CPz) electrode locations separately, in which the factors Condition (congruent, incongruent, and control) × Duration (40, 120, 200, and 300 ms) were compared to the N400 mean amplitude. A Greenhouse–Geisser correction to *p*-values was used when appropriate to decrease the risk of falsely significant results.

#### EEG Time–Frequency Analysis

Time–frequency analysis can be used to reveal event-related oscillations properties, which cannot be depicted by ERPs (Roach and Mathalon, 2008). Time–frequency analysis can represent the energy content of the EEG signal time-locked to an event in the joint time–frequency domain, in which a complex number is estimated for each time point in the time-domain signal, yielding both time and frequency domain information. According to the time–frequency decomposition, the Event-Related Spectral Perturbation [ERSP, the mean change in spectral power (in dB) compared to baseline] analysis was performed (see Makeig et al., 2004; Roach and Mathalon, 2008), particularly the ERSP of theta band activities were analyzed based on the analysis in the introduction. The eeglab 13 (Delorme and Makeig, 2004) was employed for the time–frequency analysis.

#### Source-Localization Analysis

To compare cortical source differences between EEG activities of expressions with a long duration (>200 ms, macroexpressions) and expressions with a short duration (<200 ms, microexpressions), the standardized low resolution brain electromagnetic tomography (sLORETA) software (publicly available free academic software^2^) was used to estimate the underlying source activity by an equivalent distributed linear inverse solution (Pascual-Marqui et al., 1994, 1999, 2002). sLORETA is an improvement over the previously developed tomography LORETA (Pascual-Marqui et al., 1994). LORETA solves the “inverse problem” by finding the smoothest of all solutions with no *a priori* assumptions about the number, location, or orientation of the generators. It is important to emphasize that sLORETA has no localization bias even in the presence of measurement and biological noise (Pascual-Marqui et al., 2002).

In the current implementation of sLORETA, computations were performed in a realistic head model (Fuchs et al., 2002) using the MNI152 template (Mazziotta et al., 2001), with the three-dimensional solution space restricted to cortical gray matter as determined by the probabilistic Talairach atlas (Lancaster et al., 2000). The standard electrode positions on the MNI152 scalp were taken from Jurcak et al. (2007) and Oostenveld and Praamstra (2001). The intracerebral volume was partitioned in 6239 voxels at a 5 mm spatial resolution. To find the underlying neural generator activity that was most likely responsible for the differences in the recorded scalp potentials, sLORETA calculated the current density (*A*/*m*^2^) at each voxel allocated by a dipolar source.

To find the brain regions that are most likely involved in processing expressions with different durations, we calculated difference waves by subtracting the N170/VPP for 300 ms trials from the N170/VPP for 40 ms trials during a time window of 140–200 ms. Similarly, we calculated difference waves by subtracting the N400 of incongruent trials from the N400 of congruent trials during a time window of 350–500 ms.

## Results

In the survey of the Chinese version of the Zung Self Rating Anxiety and Depression Scales [SAS, SDS, cf., Lui et al. (2009), all scores of our participants were below the critical value of 50 for SAS (mean score = 35.9, *SD* = 5.7), and the scores of all but one participant (who scored 58 and was excluded from further analysis) were under the critical value of 53 for the SDS (mean score = 41.5, *SD* = 7.4). The results of the SAS and SDS clearly demonstrated the participants’ normal mood state. All the participants reached accuracy of greater than 80% during all the recognition tasks.

### N170/VPP

#### ERPs Data

The face-sensitive potential of VPP was maximal at the central electrode sites. Therefore, electrodes Cz and CPz were selected for statistical analysis. A 2 Channel (Cz and CPz) × 4 durations (40, 120, 200, and 300 ms) × 3 valence (happiness, fear, and neutral) repeated measures analysis of variance (ANOVA) was conducted. The main effect of Channel is significant, *F*(1,11) = 5.567, *p* = 0.038, *η_p_^2^* = 0.336; the main effects of duration and valence are both significant, *F*(3,33) = 4.176, *p* = 0.037, η_p_^2^ = 0.275; *F*(2,22) = 10.412, *p* = 0.001, η_p_^2^ = 0.486. In order to better evaluate the effect of duration and valence on the N170/VPP effect, another ANOVA was conducted for Cz and CPz electrodes separately.

**FIGURE 1:**
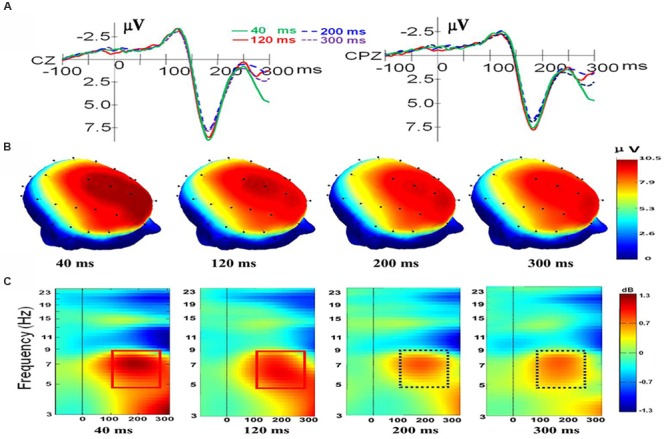
**The electroencephalogram (EEG)/event-related potentials (ERPs) results at the Cz and CPz electrode sites. (A)** The grand-averaged ERPs waveforms (N170/VPP) elicited by a fleeting facial expression with a duration of 40 (green solid), 120 (red solid), 200 (blue dashed), and 300 ms (purple dashed) at the Cz and CPz electrode sites. **(B)** Scalp potential 3D maps reveal the topography of the N170/VPP for the time window (140–200 ms). **(C)** Event-Related Spectral Perturbation (ERSP) plot showing the mean increases or decreases in spectral power following stimulation. Non-green areas in the time/frequency plane show significant (*p* < 0.01) post-stimulus increases or decreases (see color scale) in log spectral power at the CPz electrode site relative to the mean power in the averaged 1-s pre-stimulus baseline (the interval for the ERSP analysis was –1000–1500 ms).

For electrode Cz, there was a main effect of duration, *F*(3,33) = 5.027, *p* = 0.006, η_p_^2^ = 0.314. There was also a main effect of valence, *F*(2,22) = 10.824, *p* = 0.001, η_p_^2^ = 0.496, and a significant interaction was present, *F*(6,66) = 2.766, *p* = 0.018, η_p_^2^ = 0.201. Follow-up *t*-tests indicated that there is no difference between the N170/VPP mean amplitudes of happiness and fear at duration of 40 and 300 ms [*t*(11) = -0.166; *p* < 0.871; *t*(11) = -1.006; *p* < 0.336]. However, the N170/VPP mean amplitudes of happiness was bigger than that of fear at duration of 120 and 200 ms [*t*(11) = -3.612; *p* = 0.004; *t*(11) = -4.127; *p* = 0.002]. Planned comparisons of durations showed that the N170/VPP amplitude was larger for 40 ms than for 200 ms (*p* = 0.022, see **Figure 1A**). Pairwise comparisons of valence revealed that the N170/VPP amplitude was larger for fearful than for happy faces (*p* = 0.004). There was no difference between other pairings. For the electrode CPz, there was a main effect of duration, *F*(3,33) = 2.965, *p* = 0.046, η_p_^2^ = 0.212. There was also a main effect of valence, *F*(2,22) = 6.628, *p* = 0.006, η_p_^2^ = 0.376; however, there were no significant interactions, *F*(6,66) = 1.002, *p* = 0.432, η_p_^2^ = 0.083. **Figure 1** illustrates the grand average waveforms of N170/VPP at the electrodes Cz and CPz (Panel A). The scalp potential 3D maps of mean amplitude at 140–200 ms for the four corresponding levels of duration are depicted in Panel B).

#### ERSP Data

As shown in Panel C of **Figure 1**, the results of the ERSP showed that the mean post-stimulus spectral power for fleeting facial expressions with durations of 40 and 120 ms were similar (see the solid red box), and facial expressions with durations of 200 and 300 ms had a similar ERSP pattern (see the dashed purple box).

As shown in **Figure 1C**, the amplitude of theta response (4 to 8 Hz, as traditionally employed based on Berger’s studies; see Buzsáki, 2006) was higher for expressions with short duration (<200 ms) than for expressions with longer duration (>200 ms). Therefore, data of theta band activities from 100 to 260 ms of CPz were exported for performing a one-way ANOVA with repeated measures. The results showed that there was a main effect of duration, *F*(3,33) = 3.238, *p* = 0.035, η_p_^2^ = 0.227. A *post hoc* pairwise comparison of the theta response of expressions with four levels of duration showed that theta band activity of recognition for expressions with a duration of 40 ms was significantly higher than that of 200 and 300 ms (*p* = 0.006; *p* = 0.039). The comparisons found no significant difference in the theta response for pairs of expressions with durations of 40 and 120 ms or pairs of expressions with durations of 200 and 300 ms (*p* = 0.308; *p* = 0.920).

#### Source-Localization Data

**FIGURE 2:**
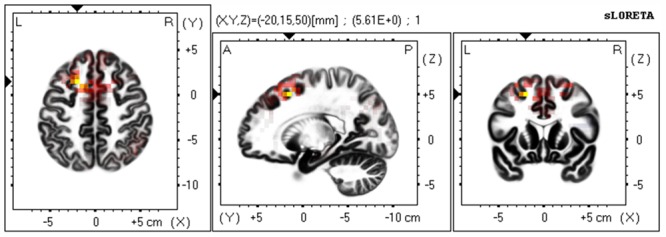
**The estimated sources of N170/VPP during a time window of 140–200 ms.** sLORETA-based statistical non-parametric maps (SnPM) comparing the standardized current density values between facial expressions with durations of 40 and 300 ms (*n* = 12) at the N170/VPP latency (140–200 ms). Significantly increased activation (*p* < 0.05) at the 40 ms duration compared to the 300 ms duration is shown in red. Each map consists of axial, sagittal, and coronal planes. The maxima are color coded as yellow. L, left; R, right; A, anterior; P, posterior.

Based on the scalp-recorded electric potential distribution, sLORETA was used to compute the cortical three-dimensional distribution of the current density of facial expressions with different durations. First, we explored standardized current density maxima for facial expressions with durations of 40, 120, 200, and 300 ms. All durations showed the same activation areas (fusiform gyrus, BA 20). To identify possible differences in the N170/VPP neural activation between the groups with durations of 40 and 300 ms, non-parametric statistical analyses of functional sLORETA images (Statistical non-Parametric Mapping; SnPM, c.f. Nichols and Holmes, 2002) were performed for the paired group while employing a *t* statistic (on log-transformed data). The results corresponded to maps of *t* statistics for each voxel, for a corrected *p* < 0.05. **Figure 2** shows sLORETA statistical non-parametric maps comparing the electric neuronal activity of recognizing expressions with durations of 40 and 300 ms at the N170/VPP latency of 140 to 200 ms. The **Figure 2** shows that the most active area of the cortex localized in the left hemisphere, in the Superior Frontal Gyrus (Brodmann area 8).

### N400

#### ERPs Data

An ANOVA on the factors of duration (40, 120, 200, and 300 ms) and congruence (congruent, incongruent, and control) was performed on the mean amplitude (350–500 ms) of the N400 to determine whether the N400 effects were influenced by the different durations.

**FIGURE 3:**
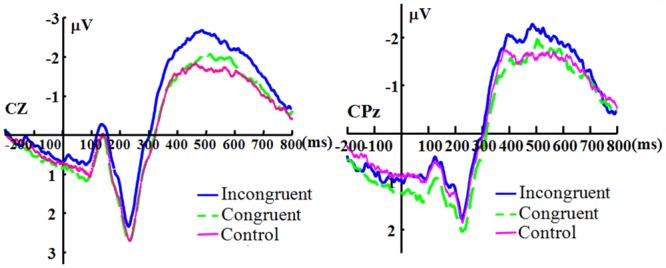
**The grand-averaged ERPs of the N400.** The grand-averaged ERPs waveforms of the N400 under the conditions of incongruence (blue solid), congruence (green dashed), and control (red) at the Cz and CPz electrode sites.

For the electrode Cz, there was no significant main effect of duration [*F*(3,33) = 2.319, *p* = 0.093, η_p_^2^ = 0.174], and the main effect of congruence was significant [*F*(2,22) = 4.503, *p* = 0.023, η_p_^2^ = 0.290]. The duration showed no significant interaction with congruence [*F*(6,66) = 1.986, *p* = 0.080, η_p_^2^ = 0.153]. For the electrode CPz, there was no significant main effect for duration [*F*(3,33) = 2.250, *p* = 0.101, η_p_^2^ = 0.170]. The main effect of congruence was significant [*F*(2,22) = 3.731, *p* = 0.040, η_p_^2^ = 0.253]. The effect of interaction of duration and congruence was not significant [*F*(6,66) = 1.142, *p* = 0.348, η_p_^2^ = 0.094]. Because there appears to be no effect of duration, **Figure 3** shows the N400 collapsed across all duration levels.

#### Source-Localization Data

**FIGURE 4:**
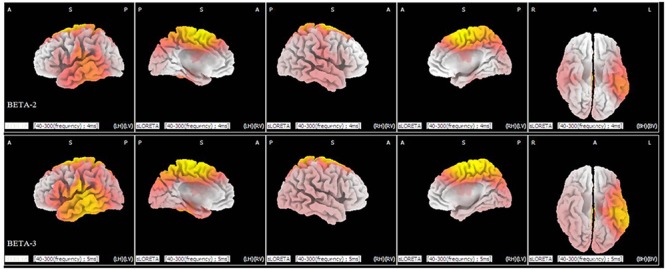
**sLORETA differences in two frequency bands (beta 2 and beta 3) between the 40 and 300 ms duration conditions (collapsed across all three congruence conditions).** In the beta 2 **(upper** panel) and beta 3 frequency bands **(lower** panel), activity was significantly higher for 40 ms than for 300 ms in widespread areas, including the medial frontal gyrus and superior frontal gyrus. Images depicting statistical parametric maps observed from different perspectives are based on voxel by-voxel log of *F*-ratio values of differences between the two groups for the beta 2 and beta 3 bands. Structural anatomy is shown in gray scale (A, anterior; P, posterior; S, superior; I, inferior; LH, left hemisphere; RH, right hemisphere; BH, both hemispheres; LV, left view; RV, right view; BV, bottom view). Yellow indicates increases for 40 ms compared to 300 ms (*t*_0.05_ = 1.314, *t*_0.01_ = 1.510, one tail), which are mainly in the medial frontal gyrus and the superior frontal gyrus.

Statistical analysis demonstrated significant differences (*p* < 0.05) between the levels of duration of 40 and 300 ms in the beta 2 (19–21 Hz) and beta 3 (22–30 Hz) frequency bands. In the beta 2 band, 371 voxels showed significant current-source density differences. In the beta 3 band, 964 voxels showed significant differences. A comparison of current density images between the 40 and 300 ms durations for beta 2 and beta 3 is shown in **Figure 4**. Yellow areas correspond to significantly higher activity in the 40 ms condition (*p* < 0.05, *t* threshold = 1.314).

## Discussion

The aim of the current study was to determine if there are different neural mechanisms underlying the recognition of microexpressions and macroexpressions. The results indicate that there are different ERPs and ERSP characteristics for recognizing microexpressions and macroexpressions. The brain regions responsible for the differences might be the inferior temporal gyrus and widespread areas in the frontal lobe. Furthermore, the left hemisphere was more involved in processing microexpressions. These results suggest that different neural mechanisms are responsible for the recognition of microexpressions and macroexpressions.

For expressions, there is a critical factor for recognition that is less well understood: the duration. A microexpression is presented for a short duration, which may result in the recipient barely perceiving it. The most commonly cited description of the duration of a microexpression: *microexpressions (1/25–1/5 of a second)*. Thus, the duration is the core difference between microexpressions and macroexpressions. Moreover, as ten Brinke and Porter (2013, p. 227) noted, a microexpression is “*a brief but complete facial expression.*” Therefore, the key characteristic differentiating microexpressions from macroexpressions is not the completeness of the expression (which may be related to intensity of emotion) but the duration of the expression. Considering that duration is the critical feature of a microexpression, in the current study, we manipulated the durations of expressions and expected that there would be different brain mechanisms for recognizing microexpressions and macroexpressions. The duration boundary may be around 200 ms, which can be used to differentiate a microexpression from a macroexpression (see Shen et al., 2012). The findings of this study show that recognizing expressions with durations of less than 200 ms and expressions with durations of greater than 200 ms are associated with different EEG/ERPs characteristics. Thus, we further confirmed that the boundary of the duration of expressions for differentiating microexpressions and macroexpressions is around 200 ms.

The present study manipulated the duration of facial expressions and examined the influence of duration on expression recognition by exploring the N170/VPP, the N400 effect and related EEG indicators. For the N170/VPP, there is a main effect of duration that clearly indicates the effects of duration on processing facial expressions with different durations. As shown in **Figure 1A**, there are two groups of ERPs, one for expressions with durations of greater than 200 ms and one for expressions with durations of less than 200 ms, suggesting that a duration boundary of 200 ms can differentiate microexpressions and macroexpressions. As for the interaction of duration and valence at electrode Cz, we should be cautious to draw any inference because there is no interaction at electrode CPz. The interaction of duration and valence should be elucidated further in the future.

As shown in **Figure 1A**, there is an enhanced N170/VPP in response to expressions with a short duration (<200 ms) compared to expressions with a long duration (>200 ms). On the one hand, the results may be due in part to attention (as a mediator variable). Attention to faces and facial expressions can modulate the N170 amplitude (Eimer, 2000, 2011; Eimer and Holmes, 2007). In the current study, recognizing expressions with short durations (e.g., 40 ms) may need significantly more attention resources (because short-duration expressions are somewhat difficult to perceive) than do expressions with long durations (e.g., 300 ms), which may result in a higher amplitude of N170/VPP for recognizing expressions with short durations. On the other hand, we automatically mimic the exposed facial expressions while recognizing them (Dimberg et al., 2000; Tamietto et al., 2009), if the exposing duration is short (say less than 200 ms, it is the case in microexpression recognition), then there is not much time to mimic the transient expression with short duration. Therefore, the mimicry of microexpression has to consult the memory to reach recognition, which may result in a stronger processing in the brain than the recognition of macroexpression, because recognizing macroexpression (with duration of greater than 200 ms) can only rely on the perceptual features of expressions.

As shown in **Figure 1**, sharp contrasts in scalp potential maps (**Figure 1B**) and ERSP (**Figure 1C**) are present between microexpressions (durations of less than 200 ms) and macroexpressions (durations of greater than 200 ms). The microexpressions elicited stronger power changes in theta band activities than did macroexpressions (see the comparison of the box of a solid line and the box of a dashed line in **Figure 1C**), which might also be interpreted as relating to the larger attention demands that are imposed on recognizing fleeting microexpressions.

In the current study, the ERSP results of N170/VPP showed that the amplitude of theta response was higher for microexpressions (with durations of less than 200 ms) than for macroexpressions (with durations of greater than 200 ms), which suggests that the theta response is also modulated by the duration of emotional expressions. Meanwhile, cognitive load may be related to the theta oscillatory activity (Bates et al., 2009). The reduced theta oscillatory activity for expressions with longer durations may be partly explained by cognitive load. For expressions with longer durations, there should be a lower cognitive load and there should be higher cognitive load for expressions with short durations. Meanwhile, the brain oscillations in the theta band are involved in active maintenance of memory representations (Jensen and Tesche, 2002). For expressions with shorter duration, one should make much more efforts to maintain the representations for further processing. For expressions with longer durations, one can check it anytime; therefore, the load for holding the representation is low. As shown in **Figure 2**, the neural generators that respond to the difference between recognizing expressions with durations of 40 and of 300 ms are located in the frontal lobe while measuring the N170/VPP, which is consistent some previous work that showed the frontal theta power increased with the cognitive load (Scheeringa et al., 2009). There are distinct EEG mu responses while viewing positively and negatively valenced emotional faces (see Moore et al., 2012), therefore, besides the beta rhythm, we should use mu response to further investigate the recognition of microexpression and macroexpression in ther future.

The statistical results of the N400 effects show no effects of duration and only a marginal significant interaction between duration and congruence at Cz, which does not support the prediction regarding the N400 effects (there should be a significant interaction). The effect of congruence is significant in the N400, which can be observed in **Figure 3** The results suggest that even under the condition of short duration of expression, the participants could engage in top-down processing and the meaning of the expression was processed regardless of the duration (long or short in the current study), which implies that the fleeting emotional expressions (even with a duration of 40 ms) can be rapidly identified at a conceptual level (Potter, 2012). The results are consistent with some previous studies (Murphy and Zajonc, 1993; Milders et al., 2008).

As shown in **Figure 4**, there are significant differences in the profiles of the beta 2 and beta 3 powers between the 40 ms duration condition and the 300 ms duration condition, which suggests a strong involvement of beta-band synchronization in the processing of duration of an expression. Beta rhythm has been observed experimentally under the conditions of extensive recruitment of excitatory neurons (Whittington et al., 2000), suggesting there are more excitatory neurons for processing a facial expression with a duration of 40 ms than there are for an expression with a duration of 300 ms. Meanwhile, from the results of the sLORETA in **Figure 4**, we can observe that there is an increase in the power of beta activities. The locations are mainly in the frontal lobe and temporal lobe and involve more left than right hemispheric voxels (a similar neural activities pattern that involve more left than right hemispheric voxels can be seen in **Figure 2**). The results suggest there is left-hemisphere dominance for recognizing microexpressions. The lateralization of emotion has long been studied (Indersmitten and Gur, 2003) and many studies show evidence supporting right-hemispheric dominance for emotion processing (Schwartz et al., 1975; Hauser, 1993). There is, however, some debate regarding right-hemispheric dominance (De Winter et al., 2015). The current results show that the left hemisphere might respond during the processing of fleeting (<200 ms) expressions, regardless of valence. The effect of duration on the hemispheric dominance for emotional expressions processing should be further investigated and some objective indexes such as weighted lateralization index (see De Winter et al., 2015) should be provided.

It should be noted that the differences between the recognition of microexpressions and macroexpressions is not the same as the differences in recognizing supraliminal and subliminal facial expression (Balconi and Lucchiari, 2005). According to Shen et al. (2012), the accuracy of recognition for expressions with durations of 40 ms is above 40%, which is higher than the chance level (1/6), which means that the recognizing microexpression is conscious. Even the expressions cannot be perceived consciously, we still can unconsciously “resonate” the facial expressions we have seen during emotion communication (Dimberg et al., 2000; Tamietto et al., 2009), which may facilitate the recognition of microexpression and macroexpression.

In summary, we wanted to determine the exact differences in neural substrates for recognizing microexpression and macroexpression in the current study. The EEG/ERPs results revealed a distinct amplitude of the N170/VPP and oscillatory neuronal dynamics in response to microexpressions (with durations of less than 200 ms) and macroexpressions (with durations of greater than 200 ms). These results suggest that the EEG/ERPs characteristics are different between the recognition of microexpressions and macroexpressions.

Our understanding of how we perceive and recognize microexpressions and macroexpressions will be further advanced by studying the EEG/ERPs, their oscillatory neuronal dynamics, and their association with the processes of recognition. Based on this understanding of microexpression recognition, we can further explore the association between microexpressions and deception. Although controversial, microexpressions are closely related to deception and are used as a vital behavioral clue for lie detection (Frank and Svetieva, 2015). According to Weinberger (2010), few published peer-review studies address microexpressions for political reasons. Linking microexpressions to deception is “a leap of gargantuan dimensions” (for a review, see Weinberger, 2010). Many more studies are needed to understand the mechanisms underlying recognition of microexpression and its association with deception.

In the future, dynamic facial expressions with greater ecological validity should be employed. The brain mechanisms involved in recognizing a number of fleeting social emotions, including shame, guilt, and remorse (ten Brinke et al., 2012), and the fundamental properties of microexpressions recognition (Svetieva, 2014) should be explored. The influence of some factors, for instance, contextual cues (Van den Stock and de Gelder, 2014) which co-occur with facial microexpresion and macroexpression, age (Zhao et al., 2016), empathy (Svetieva and Frank, 2016), on how we recognize microexpresion and macroexpression and the underlying brain mechanisms should also be investigated.

## Author Contributions

XF and XS conceived the experiments. QW and KZ conducted the experiments. XS analyzed the results and wrote the main manuscript text and prepared all figures and tables. All authors reviewed the manuscript.

## Conflict of Interest Statement

The authors declare that the research was conducted in the absence of any commercial or financial relationships that could be construed as a potential conflict of interest.

## Footnotes

1 http://www.paulekman.com

2 http://www.uzh.ch/keyinst/loreta.htm
